# Joint Resource Optimization for Cognitive Sensor Networks with SWIPT-Enabled Relay

**DOI:** 10.3390/s17092093

**Published:** 2017-09-13

**Authors:** Weidang Lu, Yuanrong Lin, Hong Peng, Tian Nan, Xin Liu

**Affiliations:** 1 College of Information Engineering, Zhejiang University of Technology, Hangzhou 310014, China; luweid@zjut.edu.cn (W.L.); zjut_lyr@163.com (Y.L.); 15757175896@163.com (T.N.); 2 School of Information and Communication Engineering, Dalian University of Technology, Dalian 116024, China; liuxinstar1984@dlut.edu.cn

**Keywords:** energy harvesting, SWIPT, CSN, amplify-and-forward, power splitting

## Abstract

Energy-constrained wireless networks, such as wireless sensor networks (WSNs), are usually powered by fixed energy supplies (e.g., batteries), which limits the operation time of networks. Simultaneous wireless information and power transfer (SWIPT) is a promising technique to prolong the lifetime of energy-constrained wireless networks. This paper investigates the performance of an underlay cognitive sensor network (CSN) with SWIPT-enabled relay node. In the CSN, the amplify-and-forward (AF) relay sensor node harvests energy from the ambient radio-frequency (RF) signals using power splitting-based relaying (PSR) protocol. Then, it helps forward the signal of source sensor node (SSN) to the destination sensor node (DSN) by using the harvested energy. We study the joint resource optimization including the transmit power and power splitting ratio to maximize CSN’s achievable rate with the constraint that the interference caused by the CSN to the primary users (PUs) is within the permissible threshold. Simulation results show that the performance of our proposed joint resource optimization can be significantly improved.

## 1. Introduction

Cognitive radio (CR) is a promising technology that aims to solve the problem of spectrum scarcity. For the underlay spectrum sharing mode, the secondary users (SUs) can share the licensed spectrum on condition that the interference to primary users (PUs) caused by the transmission of the SUs is within the permissible threshold [1]. CR can also be exploited by wireless sensor networks (WSNs), which conventionally adopt the fixed spectrum allocation over increasingly crowded unlicensed bands, and this type of cognitive networks (CNs) are called cognitive sensor networks (CSNs) [2,3].

Energy-constrained wireless networks, such as WSNs, are usually powered by fixed energy supplies (e.g., batteries), which limit the operation time of networks. Since it is not only costly but also inconvenient to replace or recharge the batteries, energy harvesting has been regarded as a feasible method that can prolong the lifetime of WSNs and becomes attractive [4]. In addition to the typical energy harvesting techniques, such as wind and solar [5,6], harvesting energy form ambient radio-frequency (RF) signals is a new emerging technique [7]. It is noted that this technique is suitable for WSNs, which is a low-power application. Meanwhile, simultaneous wireless information and power transfer (SWIPT) has drawn increasing attention since it takes advantage of RF that can carry energy and information simultaneously [8,9,10,11].

In [8], Varshney proposed the idea of SWIPT for the first time and defined a capacity-energy function to deal with the fundamental tradeoff for SWIPT. However, as discussed in [9], there is a potential limitation that the receiver cannot harvest energy and decode information from the same signal. Reference [10] investigated the application of SWIPT in wireless point-to-point communication, in which various tradeoffs between energy harvesting and information transfer were derived. Different from the traditional viewpoints, Reference [10] dealt with the interference as a new energy source. The application of SWIPT in CNs was considered in [11], where secondary transmitters harvest energy from the primary network.

Cooperative relaying based SWIPT was investigated in [12,13,14,15,16,17,18]. Reference [12] studied the energy-efficient coope rative transmission problem for clustered WSNs, in which two adjacent cluster heads communicate by one-hop energy harvesting relay. Reference [13] considered applying the SWIPT technique to the amplify-and-forward (AF) cooperative network. Reference [14] investigated the application of SWIPT in wireless cooperative networks with random relays, in which one source and multiple sources two scenarios were considered. In [15], SWIPT for relay-assisted CNs was studied, where both source and relay harvest energy from the primary user’s signal. Under three power constraints for coexisting networks, the expression for outage probability was derived. In [16], a suboptimal joint relay selection and power allocation scheme was proposed for the underlay cognitive two-way network with *L* SWIPT-enabled relays. In [17], the performance of outage probability was given in an underlay CN, where relay harvests energy using the time switching (TS) relaying protocol. However, the optimal energy harvesting duration was derived through the simulation. The approximate expressions for throughput and ergodic sum-rate of AF cognitive network with energy harvesting relay were derived in [18], while the interference caused by the relay node was ignored.

In this paper, we obtain the transmission rate expression of AF CSNs with energy harvesting relay by considering the interference caused by the relay sensor node. Moreover, the closed-form expressions for the optimal value of transmit power and power splitting ratio are derived. The main contributions of this work are summarized as follows:

- Firstly, we derive the transmission rate expression of AF CSNs with energy harvesting relay by considering the interference caused by the relay sensor node, which was ignored in [18].
- Secondly, an algorithm is proposed to obtain the closed-form optimal value of transmit power and power splitting ratio—unlike [17], in which the optimal energy harvesting duration was derived through the simulation.
- Finally, we show that there is no performance gap between our proposed algorithm and exhaustive search method.

The remainder of this paper is organized as follows. Section 2 describes the system model and the problem formulation for the underlay CSN with SWIPT-enabled relay node. An algorithm to solve the transmit power and power splitting ratio joint optimization problem is given in Section 3. Simulation results are presented in Section 4. Finally, Section 5 concludes the paper.

## 2. System Model and Problem Formulation

### 2.1. System Model

**Figure 1:**
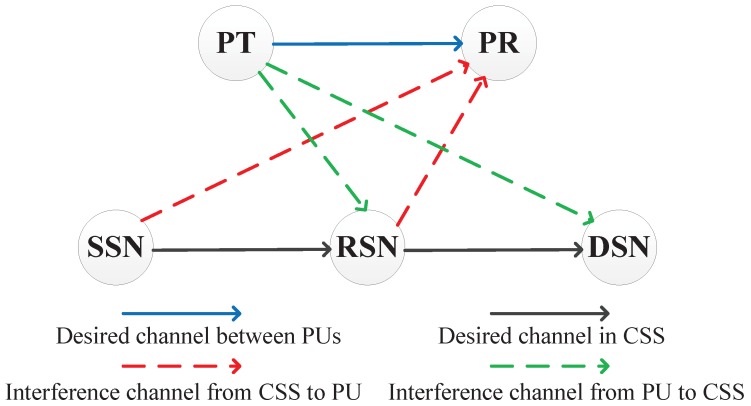
System model.

We consider an underlay CSN consisting of a primary system and a cognitive sensor system (CSS), as illustrated in Figure 1. The primary system contains a primary transmitter (PT) and a primary receiver (PR), and the CSS contains a source sensor node (SSN) and a destination sensor node (DSN). There is no direct link between SSN and DSN. Thus, the signal transmitted from SSN to DSN is forwarded by a relay sensor node (RSN).

All the terminals are equipped with one single antenna. The transmissions from CSS will cause interference to PR. At the same time, the transmit signals from PT will be treated as the interference at CSS. Between any terminals *u* and *v*, the channel coefficient is denoted as $h_{u,v}$, and $h_{u,v}=g_{u,v}d_{u,v}^{-m/2}$, where $d_{u,v}$ is the distance between *u* and *v*, and *m* is the path loss exponent, $g_{u,v}∼\mathrm{CN}\left(0,\mu \right)$ is Rayleigh fading coefficient and μ=1.

The relaying communication takes place in two equal phases. During the first phase, SSN transmits the signal to RSN, and the received signal at the RSN is expressed as

(1) $$y=\sqrt{P_{s}}x_{s}h_{\mathrm{SSN},\mathrm{RSN}}+n_{a}+\sqrt{P_{p}}x_{p}h_{\mathrm{PT},\mathrm{RSN}},$$

where $P_{s}$ and $P_{p}$ are the transmit power of SSN and PT, respectively. $x_{s}$ and $x_{p}$ are the normalized signals transmitted by the SSN and PT, respectively. $n_{a}∼\mathrm{CN}\left(0,\sigma_{a}^{2}\right)$ is the additive white Gaussian noise (AWGN) at the RSN.

The RSN splits the received signal into two parts: one part with the power splitting ratio λ (0≤λ≤1) is used for the energy harvesting and the other part (1−λ) is used for information processing. The signal for energy harvesting can be expressed as

(2) $$E=\frac{1}{2}\eta \lambda \left(P_{s}{\left|h_{\mathrm{SSN},\mathrm{RSN}}\right|}^{2}+\sigma_{a}^{2}+P_{p}{\left|h_{\mathrm{PT},\mathrm{RSN}}\right|}^{2}\right),$$

where $\eta \left(0<\eta <1\right)$ is the energy conversion efficiency. Then, the transmitted power of RSN is

(3) $$P_{r}=E/\left(1/2\right)=\eta \lambda \left(P_{s}{\left|h_{\mathrm{SSN},\mathrm{RSN}}\right|}^{2}+\sigma_{a}^{2}+P_{p}{\left|h_{\mathrm{PT},\mathrm{RSN}}\right|}^{2}\right).$$

In the second phase, RSN amplifies the received signal and forwards it to DSN. The transmitted signal of RSN is expressed as

(4) $$y_{r}=\phi \left(\sqrt{\left(1-\lambda \right)}y+n_{b}\right),$$

where $n_{b}∼\mathrm{CN}\left(0,\sigma_{b}^{2}\right)$ is the noise caused by the signal conversion from RF band to baseband [13], and ϕ is the amplification factor of RSN, which is represented as [19]

(5) $$\phi =\sqrt{\frac{P_{r}}{\left(1-\lambda \right)\left(P_{s}|h_{\mathrm{SSN},\mathrm{RSN}}{|}^{2}+\sigma_{a}^{2}+P_{p}|h_{\mathrm{PT},\mathrm{RSN}}{|}^{2}\right)+\sigma_{b}^{2}}}\approx \sqrt{\frac{\eta \lambda }{1-\lambda }}.$$

In Section 4, we demonstrate the approximation by using mathematical simulation. In the second phase, the received signal at the DSN is given by

(6) $$y_{d}=y_{r}h_{\mathrm{RSN},\mathrm{DSN}}+n_{c}+\sqrt{P_{p}}x_{p}h_{\mathrm{PT},\mathrm{DSN}},$$

where $n_{c}∼\mathrm{CN}\left(0,\sigma_{c}^{2}\right)$ is AWGN at the DSN.

Substituting Labels (1), (4) and (5) into Label(6), we can obtain

(7) $$\begin{array}{cc} y_{d} & =\sqrt{\eta \lambda P_{s}}h_{\mathrm{SSN},\mathrm{RSN}}h_{\mathrm{RSN},\mathrm{DSN}}x_{s}+\left(\sqrt{\eta \lambda P_{p}}h_{\mathrm{PT},\mathrm{RSN}}h_{\mathrm{RSN},\mathrm{DSN}}+\sqrt{P_{p}}h_{\mathrm{PT},\mathrm{DSN}}\right)x_{p}\\ & +\sqrt{\frac{\eta \lambda }{1-\lambda }}h_{\mathrm{RSN},\mathrm{DSN}}\left(\sqrt{1-\lambda }n_{a}+n_{b}\right)+n_{c}. \end{array}$$

From Label (7), we can compute the signal-to-interference-plus-noise ratio (SINR) at the DSN as the following:

(8) $$SINR=\frac{-A\lambda^{2}+A\lambda }{-B\lambda^{2}+\left(B+C-D\right)\lambda +D}P_{s},$$

where $A=\eta {\left|h_{\mathrm{SSN},\mathrm{RSN}}\right|}^{2}{\left|h_{\mathrm{RSN},\mathrm{DSN}}\right|}^{2}$, $B=\eta P_{p}{\left|h_{\mathrm{RSN},\mathrm{DSN}}\right|}^{2}{\left|h_{\mathrm{PT},\mathrm{RSN}}\right|}^{2}+\eta {\left|h_{\mathrm{RSN},\mathrm{DSN}}\right|}^{2}\sigma_{a}^{2}$, $C=\eta {\left|h_{\mathrm{RSN},\mathrm{DSN}}\right|}^{2}\sigma_{b}^{2}$ and $D=P_{p}{\left|h_{\mathrm{PT},\mathrm{DSN}}\right|}^{2}+\sigma_{c}^{2}$.

Therefore, the achievable rate at the DSN is given by

(9) $$R_{d}=\frac{1}{2}\log_{2}\left(1+SINR\right).$$

### 2.2. Problem Formulation

During the first and second phase, the interference caused by SSN and RSN to PR is given by

(10) $$I_{s}=P_{s}{\left|h_{\mathrm{SSN},\mathrm{PR}}\right|}^{2},$$

(11) $$I_{r}=P_{r}{\left|h_{\mathrm{RSN},\mathrm{PR}}\right|}^{2}=\eta \lambda \left(P_{s}{\left|h_{\mathrm{SSN},\mathrm{RSN}}\right|}^{2}+\sigma_{a}^{2}+P_{p}{\left|h_{\mathrm{PT},\mathrm{RSN}}\right|}^{2}\right){\left|h_{\mathrm{RSN},\mathrm{PR}}\right|}^{2}.$$

Thus, the optimization problem is formulated as

(12a) $$\begin{array}{cc} OP1:\;\;\; & \max_{P_{s},\lambda }\;\;R_{d}, \end{array}$$

(12b) $$\begin{array}{cc} s.t.\;\;\;\;\;\; & C1:\;I_{s}\leq I_{th}, \end{array}$$

(12c) $$\begin{array}{c} C2:\;I_{r}\leq I_{th}, \end{array}$$

(12d) $$\begin{array}{c} C3:\;0<P_{s}\leq P_{max}, \end{array}$$

(12e) $$\begin{array}{c} C4:\;\lambda \in [0,1], \end{array}$$

where C1 and C2 denote that the interference caused by CSS to PR should not be larger than $I_{th}$. C3 denotes that the maximum transmit power for SSN should not be larger than $P_{max}$. C4 shows the practical constraint of λ.

Since log(x) is a monotone increasing function of *x*, log can be omitted in the object function. Moreover, 1 is an invariant constant. Thus, OP1 can be transformed into the following problem:

(13a) $$\begin{array}{cc} OP2:\;\;\; & \max_{P_{s},\lambda }\;\;SINR, \end{array}$$

(13b) $$\begin{array}{cc} s.t.\;\;\;\;\;\; & C1,\;C2,\;C3,\;C4. \end{array}$$

## 3. Joint Optimization of Transmit Power and Power Allocation Ratio

In this part, we solve the above problem with the following two steps. Firstly, we find the optimal power splitting ratio $\lambda^{*}$ with fixed transmit power $P_{s}$. Then, we find the optimal transmit power $P_{s}^{*}$. We will show in the numerical results that there is no performance gap between the above solution with the exhaustive search.

### 3.1. Finding the $\lambda^{*}$ with Fixed $P_{s}$

Taking the first derivation of Label (8) with λ, we can obtain

(14) $$\frac{d_{SINR}}{d_{\lambda }}=\frac{A\left(D-C\right)\lambda^{2}-2AD\lambda +AD}{{\left[-B\lambda^{2}+\left(B+C-D\right)\lambda +D\right]}^{2}}P_{s}.$$

Obviously, the fact that $\frac{d_{SINR}}{d_{\lambda }}$ is positive or negative only depends on the value of $f\left(\lambda \right)=A\left(D-C\right)\lambda^{2}-2AD\lambda +AD,$ as $P_{s}$ and ${\left[-B\lambda^{2}+\left(B+C-D\right)\lambda +D\right]}^{2}$ are always positive. Moreover, it is easy to find that whether $f\left(\lambda \right)$ is positive or negative not only relies on the constraint of λ, but also the relative values of *C* and *D*. Thus, $\lambda^{*}$ can be obtained by analyzing the relative values of *C* and *D* with the constraint of λ.

**Case 1** when D<C

Apparently, $f\left(\lambda \right)$ is a quadratic function of λ, and we can find

(15) $$f\left(1\right)=A\left(D-C\right)-2AD+AD=-AC<0,$$

(16) $$f\left(0\right)=AD>0.$$

The discriminant of corresponding quadratic equation can be written as:

(17) $$\Delta ={\left(-2AD\right)}^{2}-4A\left(D-C\right)AD=4A^{2}CD>0.$$

Thus, the equation has two different roots and can be respectively written as

(18) $$\lambda_{1}=\frac{2AD-2A\sqrt{CD}}{2A\left(D-C\right)}=\frac{D-\sqrt{CD}}{D-C},$$

(19) $$\lambda_{2}=\frac{2AD+2A\sqrt{CD}}{2A\left(D-C\right)}=\frac{D+\sqrt{CD}}{D-C}.$$

From Labels (18) and (19), we can find that $0<\lambda_{1}<1$ and $\lambda_{2}<0$. From Labels (15) and (16), we can find that there is only one maxima that lies between $\left[0,1\right]$, and the position is at $\lambda =\lambda_{1}$. Thus, it is obvious that, for $\lambda <\lambda_{1}$, SINR is a monotone increasing function of λ; and, for $\lambda >\lambda_{1}$, SINR is a monotone decreasing function of λ. Meanwhile, we should consider the constraint C2. From the constraint C2, we can obtain

(20) $$\lambda \leq \lambda_{th}=\frac{I_{th}}{EP_{s}+F},$$

where $E=\eta {\left|h_{\mathrm{SSN},\mathrm{RSN}}\right|}^{2}{\left|h_{\mathrm{RSN},\mathrm{PR}}\right|}^{2}$, $F=\eta \left(\sigma_{a}^{2}+P_{p}{\left|h_{\mathrm{PT},\mathrm{RSN}}\right|}^{2}\right){\left|h_{\mathrm{RSN},\mathrm{PR}}\right|}^{2}$. Thus, if $\lambda_{th}\leq \lambda_{1}$, $\lambda^{*}=\lambda_{th}$; otherwise, $\lambda^{*}=\lambda_{1}$.

**Case 2** when D>C

With the similar analysis in **Case 1**, the optimal value of λ can be obtained as

(21) $$\lambda^{*}=\left\{\begin{array}{cc} \lambda_{1}=\frac{D-\sqrt{CD}}{D-C},\;\;\; & \mathrm{if}\;\lambda_{th}>\lambda_{1},\\ \lambda_{th}=\frac{I_{th}}{EP_{s}+F}, & \mathrm{if}\;\lambda_{th}\leq \lambda_{1}. \end{array}\right.$$

**Case 3** when D=C

Apparently, $f\left(\lambda \right)$ is a linear function of λ. We can find

(22) $$f\left(1\right)=-2AD+AD=-AD<0,$$

(23) $$f\left(0\right)=AD>0,$$

(24) $$f\left(\frac{1}{2}\right)=-2AD*\frac{1}{2}+AD=0.$$

From Labels (22)–(24), we can find that, for $\lambda <\frac{1}{2}$, SINR is a monotone increasing function of λ, and, for $\lambda >\frac{1}{2}$, SINR is a monotone decreasing function of λ. Meanwhile, we also should consider the constraint C2 as above. Then, if $\lambda_{th}\leq \frac{1}{2}$, $\lambda^{*}=\lambda_{th}$; otherwise, $\lambda^{*}=\frac{1}{2}$.

Concluded from the above analyses in **Case 1** to **Case 3**, we can obtain that when D≠C, the optimal value of λ is

(25) $$\lambda^{*}=\left\{\begin{array}{cc} \lambda_{1}=\frac{D-\sqrt{CD}}{D-C},\;\;\; & \mathrm{if}\;\lambda_{th}>\lambda_{1},\\ \lambda_{th}=\frac{I_{th}}{EP_{s}+F}, & \mathrm{if}\;\lambda_{th}\leq \lambda_{1}, \end{array}\right.$$

when D=C, the optimal value of λ is

(26) $$\lambda^{*}=\left\{\begin{array}{cc} \frac{1}{2},\;\;\; & \mathrm{if}\;\lambda_{th}>\frac{1}{2},\\ \lambda_{th}=\frac{I_{th}}{EP_{s}+F}, & \mathrm{if}\;\lambda_{th}\leq \frac{1}{2}. \end{array}\right.$$

### 3.2. Finding $P_{s}^{*}$

From Section 3.1, we can find that $\lambda^{*}$ may have three different values, which are $\lambda_{1}$, $\frac{1}{2}$ and $\lambda_{th}$. The optimal power allocation is obtained depending on the different values of $\lambda^{*}$.

**Case 1** when $\lambda^{*}=\lambda_{1}$, it should satisfy D≠C and $\lambda_{th}>\lambda_{1}$.

Substituting $\lambda^{*}=\lambda_{1}=\frac{D-\sqrt{CD}}{D-C}$ into Label (8), we can obtain

(27) $$SINR=\frac{AC+AD-2A\sqrt{CD}}{BC+BD-2CD+C^{2}+D^{2}-2B\sqrt{CD}}P_{s}=\frac{A{\left(\sqrt{C}-\sqrt{D}\right)}^{2}}{B{\left(\sqrt{C}-\sqrt{D}\right)}^{2}+{\left(C-D\right)}^{2}}P_{s}.$$

To satisfy the constraints C1, C2 and C3, we can obtain

(28) $$P_{s}\leq P_{s}^{C1}=\frac{I_{th}}{{\left|h_{\mathrm{SSN},\mathrm{PR}}\right|}^{2}},$$

(29) $$P_{s}\leq P_{s}^{C2}=\frac{\left(\frac{I_{th}\left(D-C\right)}{\eta \left(D-\sqrt{CD}\right){\left|h_{\mathrm{RSN},\mathrm{PR}}\right|}^{2}}-\sigma_{a}^{2}-P_{p}{\left|h_{\mathrm{PT},\mathrm{RSN}}\right|}^{2}\right)}{{\left|h_{\mathrm{SSN},\mathrm{RSN}}\right|}^{2}},$$

(30) $$P_{s}\leq P_{s}^{C3}=P_{max}.$$

From Label (27), we can find that SINR is a monotone increasing function of $P_{s}$. Thus, if $\min\left(P_{s}^{C1},P_{s}^{C3}\right)\geq P_{s}^{C2}$, SINR obtains the maximum value when $P_{s}=P_{s}^{C2}$. However, when $P_{s}=P_{s}^{C2}$, we can get $\lambda_{th}=\lambda_{1}$, which does not satisfy the condition that $\lambda_{1}<\lambda_{th}$. Thus, when $\lambda^{*}=\lambda_{1}$, we can get that $\min\left(P_{s}^{C1},P_{s}^{C3}\right)<P_{s}^{C2}$, and $P_{s}\in \left[0,\min\left(P_{s}^{C1},P_{s}^{C3}\right)\right]$. Thus, when $P_{s}=\min\left(P_{s}^{C1},P_{s}^{C3}\right)$, SINR obtains the maximum value. The optimal value of $P_{s}$ can be obtained as

(31) $$P_{s}^{*}=\min\left(P_{s}^{C1},P_{s}^{C3}\right).$$

**Case 2** when $\lambda^{*}=\frac{1}{2}$, it should satisfy D=C and $\lambda_{th}>\frac{1}{2}$.

With a similar analysis in **Case 1**, the optimal value of $P_{s}$ can be obtained as

(32) $$P_{s}^{*}=\min\left(P_{s}^{C1},P_{s}^{C3}\right).$$

**Case 3** when $\lambda^{*}=\lambda_{th}$

From Labels (25) and (26), we can find that, in this case, it will have two different conditions.

**Condition 1** D≠C and $\lambda_{th}\leq \lambda_{1}$

Substituting $\lambda =\lambda_{th}=\frac{I_{th}}{EP_{s}+F}$ into Label (8), we can get

(33) $$SINR=\frac{-A{\left(\frac{I_{th}}{EP_{s}+F}\right)}^{2}+A\frac{I_{th}}{EP_{s}+F}}{-B{\left(\frac{I_{th}}{EP_{s}+F}\right)}^{2}+\left(B+C-D\right)\frac{I_{th}}{EP_{s}+F}+D}P_{s}=\frac{\alpha P_{s}^{2}+\beta P_{s}}{\gamma P_{s}^{2}+\theta P_{s}+\omega },$$

where $\alpha =AEI_{th}$, $\beta =AFI_{th}-AI_{th}^{2}$, $\gamma =DE^{2}$, $\theta =2DEF+\left(B+C-D\right)EI_{th}$, and $\omega =DF^{2}+\left(B+C-D\right)FI_{th}-BI_{th}^{2}$.

We can find that the constraint C2 is satisfied when $\lambda^{*}=\lambda_{th}$. Moreover, to satisfy constraints C1 and C3, we can obtain

(34) $$P_{s}\leq P_{s}^{C1}=\frac{I_{th}}{{\left|h_{\mathrm{SSN},\mathrm{PR}}\right|}^{2}},$$

(35) $$P_{s}\leq P_{s}^{C3}=P_{max}.$$

To satisfy the condition $\lambda_{th}\leq \lambda_{1}$, we can obtain

(36) $$P_{s}\geq P_{s}^{C4}=\frac{\left(\frac{I_{th}\left(D-C\right)}{\eta \left(D-\sqrt{CD}\right){\left|h_{\mathrm{RSN},\mathrm{PR}}\right|}^{2}}-\sigma_{a}^{2}-P_{p}{\left|h_{\mathrm{PT},\mathrm{RSN}}\right|}^{2}\right)}{{\left|h_{\mathrm{SSN},\mathrm{RSN}}\right|}^{2}}.$$

If $\min\left(P_{s}^{C1},P_{s}^{C3}\right)<P_{s}^{C4}$, then, $P_{s}\leq \min\left(P_{s}^{C1},P_{s}^{C3}\right)<P_{s}^{C4}$, which does not satisfy the condition $P_{s}\geq P_{s}^{C4}$. Thus, when $\lambda^{*}=\lambda_{th}$, we can obtain that $\min\left(P_{s}^{C1},P_{s}^{C3}\right)\geq P_{s}^{C4}$. Then, we can obtain $P_{s}\in \left[P_{s}^{C4},\min\left(P_{s}^{C1},P_{s}^{C3}\right)\right]$.

Take the first derivation of Label (33) with $P_{s}$, we can obtain

(37) $$\frac{d_{SINR}}{d_{P_{s}}}=\frac{\left(2\alpha P_{s}+\beta \right)\left(\gamma P_{s}^{2}+\theta P_{s}+\omega \right)-\left(\alpha P_{s}^{2}+\beta P_{s}\right)\left(2\gamma P_{s}+\theta \right)}{{\left(\gamma P_{s}^{2}+\theta P_{s}+\iota \right)}^{2}}=\frac{\left(\alpha \theta -\beta \gamma \right)P_{s}^{2}+2\alpha \omega P_{s}+\beta \omega }{{\left(\gamma P_{s}^{2}+\theta P_{s}+\omega \right)}^{2}}.$$

Obviously, the fact that $\frac{d_{SINR}}{d_{P_{s}}}$ is positive or negative only depends on the value of $f\left(P_{s}\right)=\left(\alpha \theta -\beta \gamma \right)P_{s}^{2}+2\alpha \omega P_{s}+\beta \omega$. $f\left(P_{s}\right)$ is a quadratic function of $P_{s}$. We can find

(38) $$\alpha \theta -\beta \gamma =AEI_{th}\left[2DEF+\left(B+C-D\right)EI_{th}\right]-\left(AFI_{th}-AI_{th}^{2}\right)DE^{2}=ADE^{2}FI_{th}+A\left(B+C\right)E^{2}I_{th}^{2}>0.$$

Thus, the opening of $f\left(P_{s}\right)$ is up. The symmetry axis can be expressed as

(39) $$P_{s}=-\frac{2\alpha \omega }{2\left(\alpha \theta -\beta \gamma \right)}=-\frac{\alpha \omega }{\alpha \theta -\beta \gamma }.$$

The discriminant of corresponding quadratic equation can be written as

(40) $$\Delta ={\left(2\alpha \omega \right)}^{2}-4\left(\alpha \theta -\beta \gamma \right)\beta \omega =4A^{2}CE^{2}I_{th}^{4}\omega .$$

We can find that the shape of $f\left(P_{s}\right)$ is related to the value of ω.

**Figure 2:**
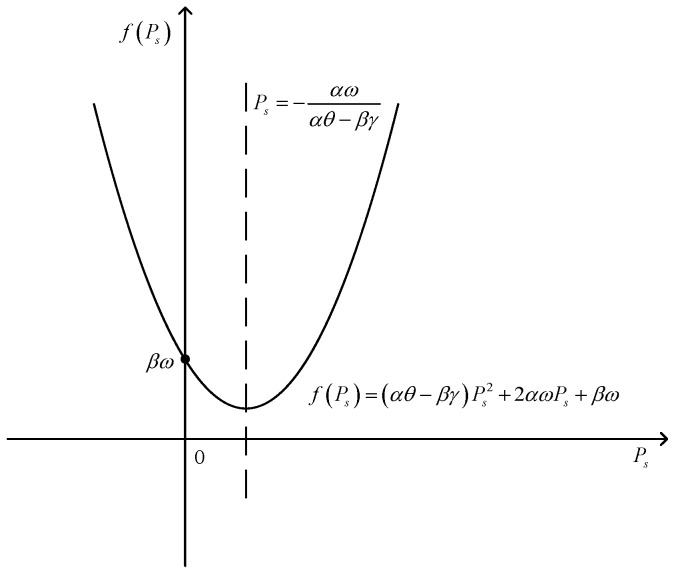
The image of $f\left(P_{s}\right)$ when ω≤0.

(1) when ω≤0, $f\left(P_{s}\right)$ is shown in Figure 2.

In this situation, $-\frac{\alpha \omega }{\alpha \theta -\beta \gamma }\geq 0$, the symmetry axis is at a nonnegative axle. If $\beta =AFI_{th}-AI_{th}^{2}\geq 0$, we can obtain $F\geq I_{th}$, then $\omega =DF^{2}+\left(B+C-D\right)FI_{th}-BI_{th}^{2}\geq CI_{th}>0$, which will not satisfy the condition that ω≤0. Thus, $\beta =AFI_{th}-AI_{th}^{2}<0$, and then we can obtain $\Delta =4A^{2}CE^{2}I_{th}^{4}\omega \leq 0$. In addition, we can obtain βω≥0. Thus, we can find that there is one zero point or none. Thus, when ω≤0, we can obtain $f\left(P_{s}\right)\geq 0$, $\frac{d_{SINR}}{d_{P_{s}}}\geq 0$, SINR is a monotone increasing function of $P_{s}$. When $P_{s}=\min\left(P_{s}^{C1},P_{s}^{C3}\right)$, SINR obtains the maximum value. Thus, the optimal value of $P_{s}$ can be obtained as

(41) $${P_{s}}^{*}=\min\left(P_{s}^{C1},P_{s}^{C3}\right).$$

(2) when ω>0

In this situation, $-\frac{\alpha \omega }{\alpha \theta -\beta \gamma }<0$, the symmetry axis is at the negative axle. Moreover, $\Delta =4A^{2}CE^{2}I_{th}^{4}\omega >0$, there are two zero points, which are $P_{s}^{1}=\frac{-\alpha \omega +\sqrt{4A^{2}CE^{2}I_{th}^{4}\omega }}{2\left(\alpha \theta -\beta \gamma \right)}$ and $P_{s}^{2}=\frac{-\alpha \omega -\sqrt{4A^{2}CE^{2}I_{th}^{4}\omega }}{2\left(\alpha \theta -\beta \gamma \right)}$, respectively. However, the shape of function $f\left(P_{s}\right)$ is also related to the value of β.

**Figure 3:**
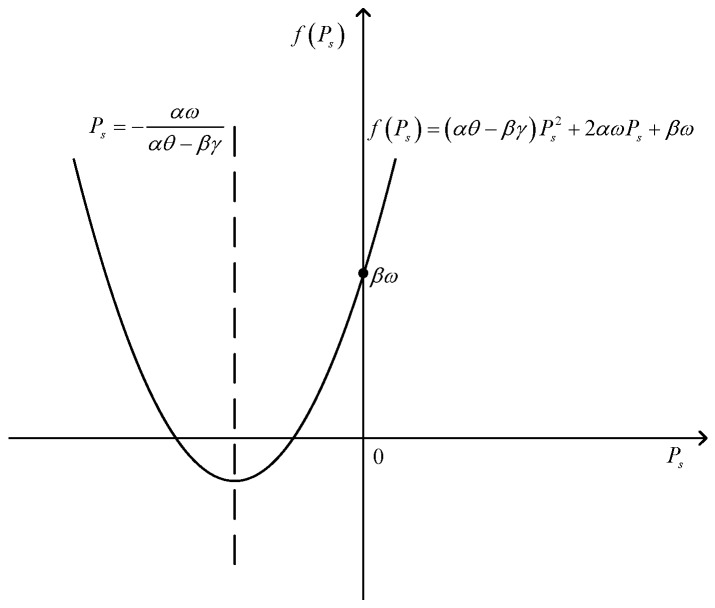
The image of $f\left(P_{s}\right)$ when ω>0, β≥0.

(i) when β≥0, $f\left(P_{s}\right)$ is shown in Figure 3.

In this situation, βω≥0, we can obtain $f\left(P_{s}\right)\geq 0$, $\frac{d_{SINR}}{d_{P_{s}}}\geq 0$, SINR is a monotone increasing function of $P_{s}$. Thus, when $P_{s}=\min\left(P_{s}^{C1},P_{s}^{C3}\right)$, SINR obtains the maximum value. Thus, the optimal value of $P_{s}$ can be obtained as

(42) $${P_{s}}^{*}=\min\left(P_{s}^{C1},P_{s}^{C3}\right).$$

(ii) when β<0

**Figure 4:**
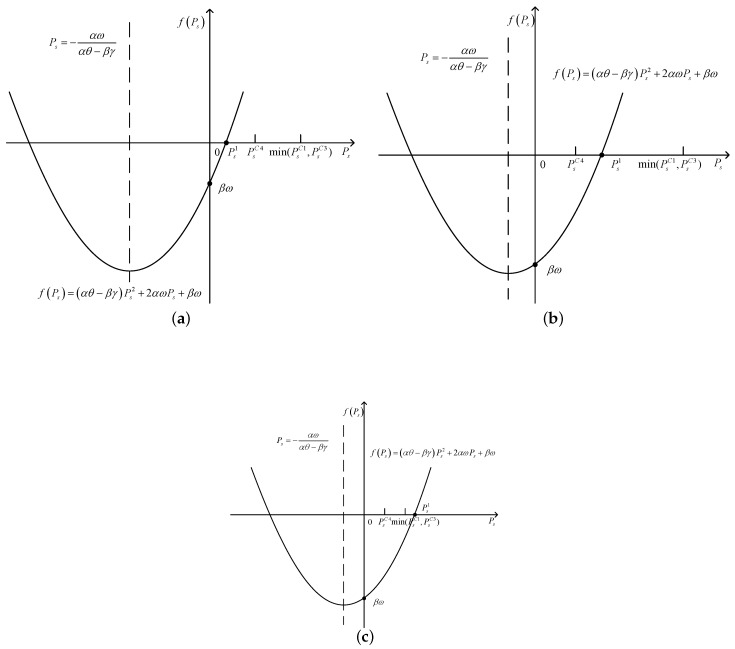
The image of $f\left(P_{s}\right)$ when ω>0, β<0. (**a**) the image of $f\left(P_{s}\right)$ when ω>0, β<0 and $P_{s}^{1}<P_{s}^{C4}$; (**b**) the image of $f\left(P_{s}\right)$ when ω>0, β<0 and $P_{s}^{C4}\leq P_{s}^{1}\leq \min\left(P_{s}^{C1},P_{s}^{C3}\right)$; (**c**) the image of $f\left(P_{s}\right)$ when ω>0, β<0 and $\min\left(P_{s}^{C1},P_{s}^{C3}\right)<P_{s}^{1}$.

In this situation, βω<0. When $P_{s}^{1}<P_{s}^{C4}$, as shown in Figure 4a, we can obtain $f\left(P_{s}\right)>0$, $\frac{d_{SINR}}{d_{P_{s}}}>0$, and SINR is a monotone increasing function of $P_{s}$. Thus, when $P_{s}=\min\left(P_{s}^{C1},P_{s}^{C3}\right)$, SINR obtains the maximum value. Thus, the optimal value of $P_{s}$ can be obtained as

(43) $${P_{s}}^{*}=\min\left(P_{s}^{C1},P_{s}^{C3}\right),$$

when $P_{s}^{C4}\leq P_{s}^{1}\leq \min\left(P_{s}^{C1},P_{s}^{C3}\right)$, as shown in Figure 4b. When $P_{s}\in \left[P_{s}^{C4},P_{s}^{1}\right]$, we can obtain $f\left(P_{s}\right)\leq 0$, $\frac{d_{SINR}}{d_{P_{s}}}\leq 0$, SINR is a monotone decreasing function of $P_{s}$; when $P_{s}\in \left(P_{s}^{1},\min\left(P_{s}^{C1},P_{s}^{C3}\right)\right]$, we can obtain $f\left(P_{s}\right)>0$, $\frac{d_{SINR}}{d_{P_{s}}}>0$, SINR is a monotone increasing function of $P_{s}$. Thus, the optimal value of $P_{s}$ can be obtained as

(44) $$P_{s}^{*}=\left\{\begin{array}{cc} \min\left(P_{s}^{C1},P_{s}^{C3}\right),\;\; & \mathrm{if}\;SINR\left(\min\left(P_{s}^{C1},P_{s}^{C3}\right)\right)\geq SINR\left(P_{s}^{C4}\right),\\ P_{s}^{C4}, & \mathrm{if}\;SINR\left(\min\left(P_{s}^{C1},P_{s}^{C3}\right)\right)<SINR\left(P_{s}^{C4}\right), \end{array}\right.$$

when $\min\left(P_{s}^{C1},P_{s}^{C3}\right)<P_{s}^{1}$, as shown in Figure 4c, we can obtain $f\left(P_{s}\right)<0$, $\frac{d_{SINR}}{d_{P_{s}}}<0$, SINR is a monotone decreasing function of $P_{s}$. Thus, when $P_{s}=P_{s}^{C4}$, SINR obtains the maximum value. Thus, the optimal value of $P_{s}$ can be obtained as

(45) $${P_{s}}^{*}=P_{s}^{C4}.$$

**Condition 2** D=C and $\lambda_{th}\leq \frac{1}{2}$

To satisfy the condition $\lambda_{th}\leq \frac{1}{2}$, we can obtain

(46) $$P_{s}\geq P_{s}^{C5}=\frac{\frac{2I_{th}}{\eta {\left|h_{\mathrm{RSN},\mathrm{PR}}\right|}^{2}}-\sigma_{a}^{2}-P_{p}{\left|h_{\mathrm{PT},\mathrm{RSN}}\right|}^{2}}{{\left|h_{\mathrm{SSN},\mathrm{RSN}}\right|}^{2}}.$$

Then, with the similar analysis in **Condition 1**, we can obtain

(47) $$P_{s}^{*}=\left\{\begin{array}{c} P_{s}^{C5},\;\;\mathrm{if}\;\left\{\begin{array}{c} 1.\omega >0,\beta <0,P_{s}^{C5}\leq P_{s}^{1}\leq \min\left(P_{s}^{C1},P_{s}^{C3}\right)\;\mathrm{and}SINR\left(\min\left(P_{s}^{C1},P_{s}^{C3}\right))<SINR(P_{s}^{C5},\right)\\ 2.\omega >0,\beta <0\;\mathrm{and}\min\left(P_{s}^{C1},P_{s}^{C3}\right)<P_{s}^{1}, \end{array}\right.\\ \min\left(P_{s}^{C1},P_{s}^{C3}\right),\;\;\mathrm{otherwise}. \end{array}\right.$$

Concluded from the above analyses in **Case 1** to **Case 3**, we can obtain that when $\lambda^{*}=\lambda_{1}$ or $\lambda^{*}=\frac{1}{2}$, the optimal value of $P_{s}$ is

(48) $${P_{s}}^{*}=\min\left(P_{s}^{C1},P_{s}^{C3}\right),$$

when $\lambda^{*}=\lambda_{th}$,

(i) if D≠C, the optimal value of $P_{s}$ is

(49) $$P_{s}^{*}=\left\{\begin{array}{c} P_{s}^{C4},\;\;\mathrm{if}\;\left\{\begin{array}{c} 1.\omega >0,\beta <0,P_{s}^{C4}\leq P_{s}^{1}\leq \min\left(P_{s}^{C1},P_{s}^{C3}\right)\;\mathrm{and}SINR\left(\min\left(P_{s}^{C1},P_{s}^{C3}\right))<SINR(P_{s}^{C4},\right)\\ 2.\omega >0,\beta <0\;\mathrm{and}\min\left(P_{s}^{C1},P_{s}^{C3}\right)<P_{s}^{1}, \end{array}\right.\\ \min\left(P_{s}^{C1},P_{s}^{C3}\right),\;\;\mathrm{otherwise} \end{array}\right.$$

(ii) if D=C, the optimal value of $P_{s}$ is

(50) $$P_{s}^{*}=\left\{\begin{array}{c} P_{s}^{C5},\;\;\mathrm{if}\;\left\{\begin{array}{c} 1.\omega >0,\beta <0,P_{s}^{C5}\leq P_{s}^{1}\leq \min\left(P_{s}^{C1},P_{s}^{C3}\right)\;\mathrm{and}SINR\left(\min\left(P_{s}^{C1},P_{s}^{C3}\right))<SINR(P_{s}^{C5},\right)\\ 2.\omega >0,\beta <0\;\mathrm{and}\min\left(P_{s}^{C1},P_{s}^{C3}\right)<P_{s}^{1}, \end{array}\right.\\ \min\left(P_{s}^{C1},P_{s}^{C3}\right),\;\;\mathrm{otherwise} \end{array}\right.$$

Based on the above analysis, Algorithm 1 presents the process of the proposed algorithm for joint optimization problem.
**Algorithm 1** Proposed Algorithm for Joint Optimization Problem.1.**if**
D≠C2. **if**
$\lambda_{th}>\lambda_{1}$
**then**3.  $\lambda^{*}=\lambda_{1}$ and $P_{s}^{*}=\min\left(P_{s}^{C1},P_{s}^{C3}\right)$4. **else**5.  **if**
$\omega >0,\beta <0,P_{s}^{C4}\leq P_{s}^{1}\leq \min\left(P_{s}^{C1},P_{s}^{C3}\right),SINR\left(\min\left(P_{s}^{C1},P_{s}^{C3}\right))<SINR(P_{s}^{C4}\right)$ or $\omega >0,\beta <0,\min\left(P_{s}^{C1},P_{s}^{C3}\right)<P_{s}^{1}$
**then**6.   $\lambda^{*}=\lambda_{th}$ and $P_{s}^{*}=P_{s}^{C4}$ substituting $P_{s}^{*}$ into $\lambda_{th}$ obtains the optimal value of $\lambda^{*}$7.  **else**8.   $\lambda^{*}=\lambda_{th}$ and $P_{s}^{*}=\min\left(P_{s}^{C1},P_{s}^{C3}\right)$ substituting $P_{s}^{*}$ into $\lambda_{th}$ obtains the optimal value of $\lambda^{*}$9.  **end if**10. **end if**11.**else**12. **if**
$\lambda_{th}>\frac{1}{2}$
**then**13.  $\lambda^{*}=\frac{1}{2}$ and $P_{s}^{*}=\min\left(P_{s}^{C1},P_{s}^{C3}\right)$14. **else**15.  **if**
$\omega >0,\beta <0,P_{s}^{C5}\leq P_{s}^{1}\leq \min\left(P_{s}^{C1},P_{s}^{C3}\right),SINR\left(\min\left(P_{s}^{C1},P_{s}^{C3}\right))<SINR(P_{s}^{C5}\right)$ or $\omega >0,\beta <0,\min\left(P_{s}^{C1},P_{s}^{C3}\right)<P_{s}^{1}$
**then**16.   $\lambda^{*}=\lambda_{th}$ and $P_{s}^{*}=P_{s}^{C5}$ substituting $P_{s}^{*}$ into $\lambda_{th}$ obtains the optimal value of $\lambda^{*}$17.  **else**18.   $\lambda^{*}=\lambda_{th}$ and $P_{s}^{*}=\min\left(P_{s}^{C1},P_{s}^{C3}\right)$ substituting $P_{s}^{*}$ into $\lambda_{th}$ obtains the optimal value of $\lambda^{*}$19.  **end if**20. **end if**21.**end if**

## 4. Simulation Results and Discussion

Unless otherwise stated, we assume that the path loss exponent m=3, the energy harvesting efficiency η=0.8, the distance $d_{\mathrm{SSN},\mathrm{RSN}}+d_{\mathrm{RSN},\mathrm{DSN}}=2$, and $d_{\mathrm{PT},\mathrm{RSN}}=d_{\mathrm{PT},\mathrm{DSN}}=d_{\mathrm{SSN},\mathrm{PR}}=d_{\mathrm{RSN},\mathrm{PR}}=\;2$, the PT transmission power $P_{p}=2W$, the maximal SSN transmission power $P_{max}=2W$. For simplicity, noise variances $\sigma_{a}^{2}=\sigma_{b}^{2}=\sigma_{c}^{2}=0.01$. All simulation results are averaged over 10,000 channel realizations.

**Figure 5:**
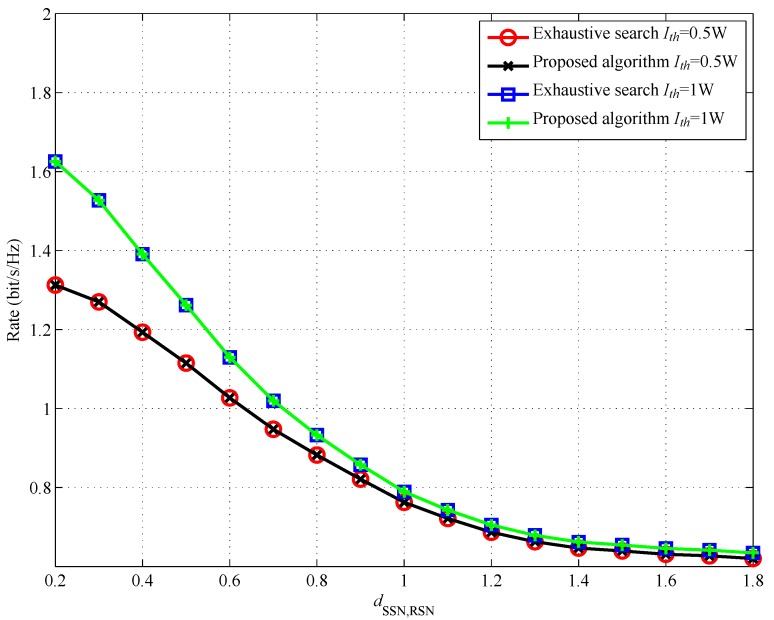
Achievable rate versus $d_{\mathrm{SSN},\mathrm{RSN}}$.

**Figure 6:**
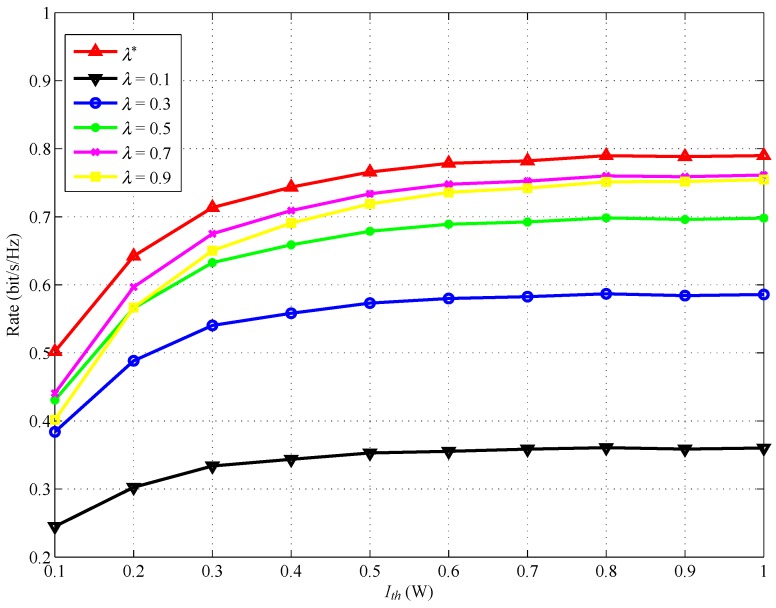
Achievable rate for various λ versus $I_{th}$.

**Figure 7:**
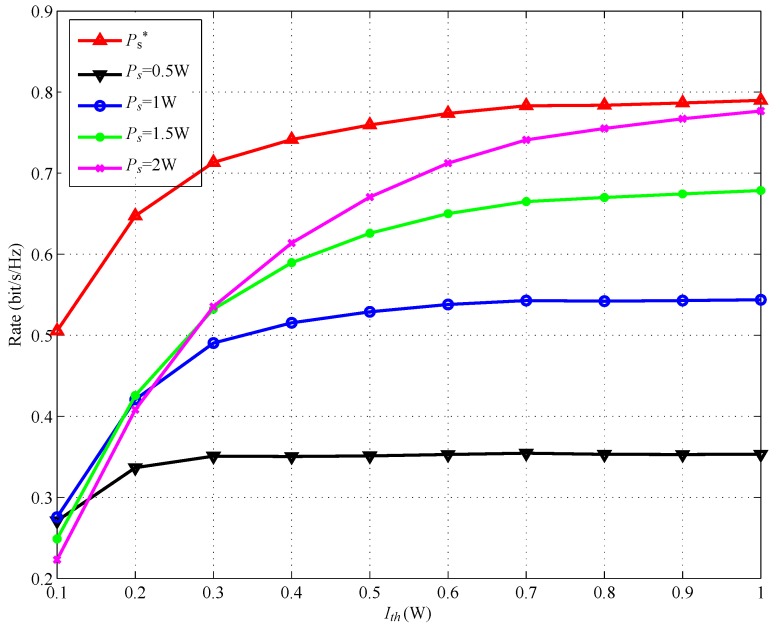
Achievable rate for various $P_{s}$ versus $I_{th}$.

Figure 5 shows the achievable rate of the cognitive sensor system versus $d_{\mathrm{SSN},\mathrm{RSN}}$. It can be observed from Figure 5 that there is no performance gap between our proposed algorithm and the exhaustive search method. In Figure 5, we can find that the achievable rate becomes larger when RSN moves closer to SSN, which is due to the fact that when RSN is located closer to SSN, it can harvest more power in the first slot to help forward SSN’s signal to DSN in the second slot. We can also observe from Figure 5 that, with larger $I_{th}$, SSN can achieve a larger rate. This is because, with a larger $I_{th}$, PR can tolerate a larger interference from the cognitive sensor network. Then, SSN and RSN can use more power to transmit the signal, which can be illustrated in Figure 6 and Figure 7.

Figure 6 and Figure 7 show the achievable rate versus $I_{th}$ with different power splitting ratio λ and with different transmit power $P_{s}$, respectively. The distance between SSN and RSN, $d_{\mathrm{SSN},\mathrm{RSN}}$, is set to be 1. In Figure 6, we can find that SSN obtains the maximum rate when RSN uses the optimal power splitting ratio $\lambda^{*}$. In Figure 7, we can also observe that SSN will obtain the maximum rate when RSN uses the optimal transmit power $P_{s}^{*}$.

**Figure 8:**
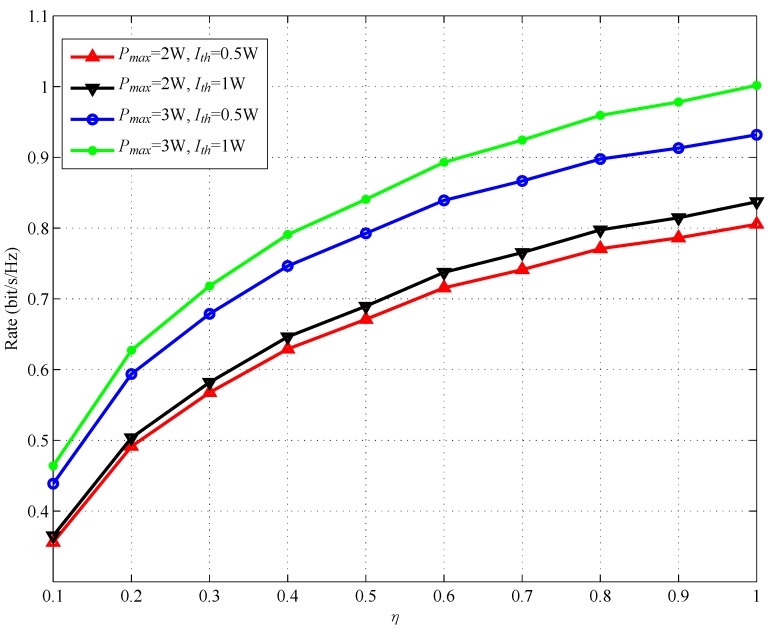
Achievable rate versus η.

**Figure 9:**
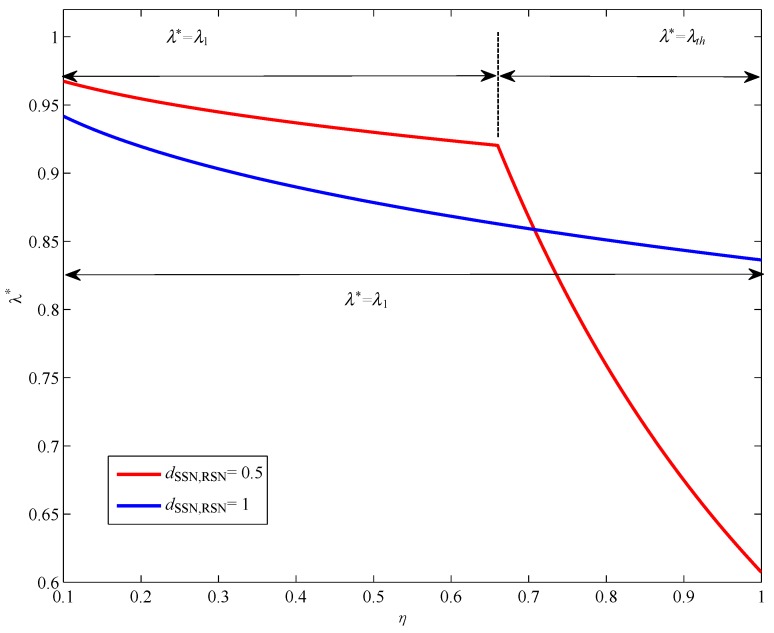
The optimal value of λ versus η.

Figure 8 shows the achievable rate versus η with different $I_{th}$ and $P_{max}$. The distance between SSN and RSN, $d_{\mathrm{SSN},\mathrm{RSN}}$, is set to be 1. In Figure 8, we can find that the achievable rate increases with the η. This is due to the fact that, with larger η, RSN can harvest more energy to help forward the SSN information, which leads to a larger achievable rate at DSN. Figure 9 shows the optimal λ versus η with different $d_{\mathrm{SSN},\mathrm{RSN}}$. The interference threshold $I_{th}$ is set to be 1 W. It can be observed from Figure 9 that $\lambda^{*}$ decreases with the increase of η. Then, RSN can use more energy to forward SSN information, which also illustrates a larger achievable rate as shown in Figure 8.

**Figure 10:**
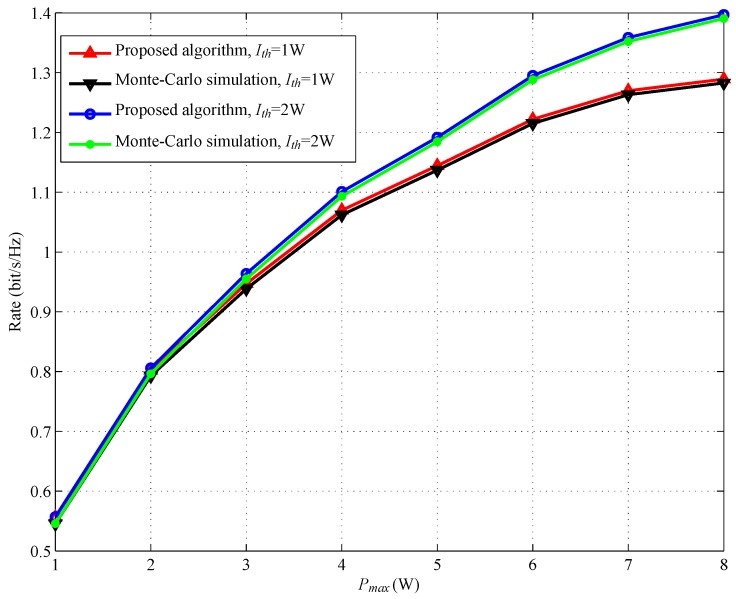
Achievable rate versus $P_{\max}$.

Figure 10 shows the achievable rate versus $P_{max}$ with different $I_{th}$. It can be observed from Figure 10 that the performance gap between the proposed algorithm and Monte Carlo simulation is very small, which illustrates the approximation in Label (5). We also can observe from Figure 10 that the achievable rate increases with the maximal SSN transmission power $P_{max}$.

**Figure 11:**
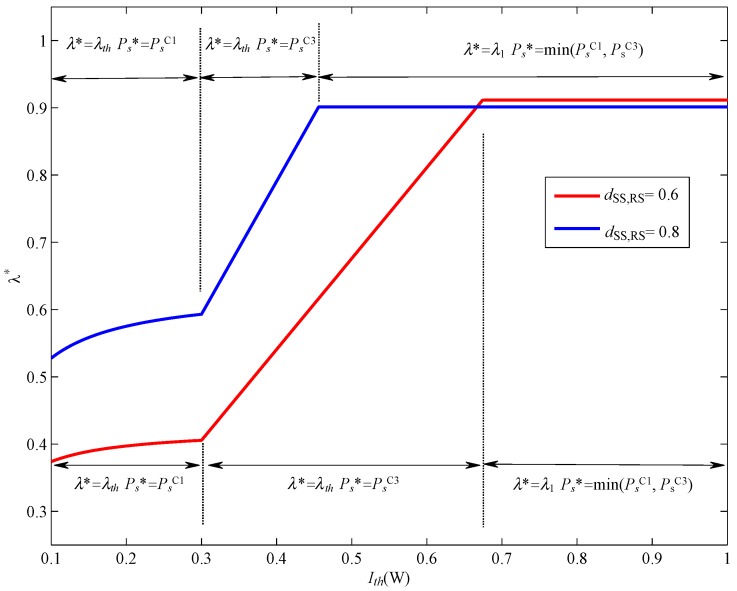
The optimal value of λ versus $I_{th}$.

Figure 11 shows the optimal λ versus $I_{th}$ with different $d_{\mathrm{SSN},\mathrm{RSN}}$. The maximal transmit power of SSN, $P_{max}$ is set to be 3W, the PT transmission power $P_{p}=3W$. It can be observed from Figure 11 that $\lambda^{*}$ becomes larger with the increase of $I_{th}$. When $I_{th}$ is sufficiently small, and we can find that $P_{s}^{C1}\leq P_{s}^{C3}$, and $\lambda^{*}=\lambda_{th}$, ${P_{s}}^{*}=P_{s}^{C1}$. As $I_{th}$ increases, $P_{s}^{C1}$ becomes larger, when $P_{s}^{C1}>P_{s}^{C3}$, we can find that $\lambda^{*}=\lambda_{th}$, ${P_{s}}^{*}=P_{s}^{C3}$, and $\lambda^{*}$ linearly increases with the increase of $I_{th}$. However, when $I_{th}$ reaches a certain level, $\lambda^{*}$ becomes to be a constant, which equals $\lambda_{1}$.

## 5. Conclusions

In this paper, we investigated the performance of underlay CSNs with SWIPT-enabled relay node. Considering the constraints of interference power at PR and the transmit power at SSN, we propose an algorithm to solve the transmit power and the power splitting ratio joint optimization problem. Meanwhile, the closed-form expressions for $\lambda^{*}$ and $P_{s}^{*}$ are derived. It is shown that there is no performance gap between our proposed algorithm and the exhaustive search method, while the complexity can be reduced.
